# The Role of Religious Coping and Preparedness in Death Acceptance Among Dementia Caregivers

**DOI:** 10.1093/geroni/igaf122.2859

**Published:** 2025-12-31

**Authors:** Landon Peeples, Lauren Chrzanowski, Benjamin Mast

**Affiliations:** University of Louisville, Louisville, Kentucky, United States; University of Louisville, Louisville, Kentucky, United States; University of Louisville, Louisville, Kentucky, United States

## Abstract

Religious coping is an important factor in how individuals manage aging and mortality, yet the mechanisms by which it influences acceptance of death are unclear. This study examines two competing path models assessing the relationships between positive and negative religious coping, death preparedness, and death acceptance. Data from 41 caregivers of older adults with dementia from the Resources for Enhancing Alzheimer’s Caregiver Health (REACH-II) dataset were analyzed to determine the best-fitting model. The first model proposed that preparedness predicts both positive and negative religious coping, which then predict acceptance. This model showed poor fit (χ² (1) = 11.393, p = .001, CFI = 0.778, TLI = -0.335), suggesting that it did not represent the data well. Preparedness significantly predicted positive (β = .554, p < .001) and negative religious coping (β = .510, p < .001), yet neither mechanism was a strong predictor of acceptance (positive: β = .281, p = .068; negative: β = .287, p = .063). Alternatively, the second model tested if religious coping predicts preparedness, which in turn predicts acceptance. This model revealed a strong fit (χ² (2) = 0.976, p = .614, CFI = 1.000, TLI = 1.000). Both positive (β = .403, p = .004) and negative (β = .322, p = .020) religious coping significantly predicted preparedness, which strongly predicted acceptance (β = .639, p < .001). Findings suggest that religious coping facilitates preparedness and indirectly acceptance, stressing the need for interventions that enhance preparedness to foster greater acceptance of death.

